# Utility of circulating tumor DNA in secondary liver malignancies: What we know and what is to come

**DOI:** 10.1002/jso.27838

**Published:** 2024-08-19

**Authors:** Chase J. Wehrle, Noah X. Tocci, Keyue Sun, Chunbao Jiao, Hanna Hong, Abby Gross, Erlind Allkushi, Melis Uysal, Maureen Whitsett Linganna, Katheryn Stackhouse, Koji Hashimoto, Andrea Schlegel, R. Matthew Walsh, Charles Miller, David C. H. Kwon, Federico Aucejo

**Affiliations:** ^1^ Department of Hepato‐Pancreato‐Biliary & Liver Transplant Surgery Cleveland Clinic Foundation, Digestive Diseases and Surgery Institute Cleveland Ohio USA; ^2^ Cleveland Clinic Foundation, Lerner Research Institute, Inflammation & Immunity Cleveland Ohio USA; ^3^ Department of Gastroenterology, Hepatology, and Nutrition Cleveland Clinic Foundation, Digestive Diseases and Surgery Institute Cleveland Ohio USA

**Keywords:** colorectal metastasis, ctDNA, liquid biopsy, liver malignancy, secondary liver malignancy

## Abstract

Secondary liver malignancies are a serious and challenging global health concern. Secondary metastasis to the liver is most commonly from colorectal cancer that has metastatically spread through splanchnic circulation. Metastatic diseases can portend poor prognosis due to the progressive nature typically found on detection. Improvements in detection of disease, monitoring therapy response, and monitoring for recurrence are crucial to the improvement in the management of secondary liver malignancies. Assessment of ctDNA in these patient populations poses an opportunity to impact the management of secondary liver malignancies. In this review, we aim to discuss ctDNA, the current literature, and future directions of this technology within secondary liver malignancies.

## 1 INTRODUCTION

Secondary liver malignancies arise from various tumors that are nonliver primary tumors in nature that have metastasized to the liver. These metastases can originate from a diverse range of primary tumors, including colorectal, breast, lung, pancreatic, and gastric cancers, among others.^1^ Secondary liver malignancies, represent a significant clinical challenge in oncology due to the inherent extensive nature of the metastatic disease and they therefore pose a global health concern. Secondary liver cancers are more common than their primary counterparts. Colorectal metastases comprise the majority of secondary malignancies. While early‐stage colorectal cancer liver metastasis (CRLM) may be treated with curative intent, advanced disease can lead to a 5‐year survival as poor as 11%.^2^ Further, only about 20% of CRLM cases are resectable at presentation.^3^, ^4^ A large contributor to the overall poor prognosis of these disease states relates to the advanced stage of disease that these cancers are often identified.^5^ This late‐stage identification is often due to indolent symptom development and the challenge of proactive detection to screen for these diseases.

Beyond colorectal cancer, secondary liver malignancies can originate from various primary tumors, including breast, lung, pancreatic, gastric, and others. For instance, breast cancer is known to metastasize to the liver in approximately 30% of patients with advanced disease.^6^ Similarly, lung cancer frequently gives rise to liver metastases, with an incidence ranging from 3% to 55% depending on the histological subtype.^7^, ^8^, ^9^

The management of secondary liver malignancies hinges on accurate diagnosis, prognostication, and treatment selection predicated by the biology of the primary tumor. Tissue sampling plays a key role in the assessment of secondary liver malignancies, however, tissue diagnosis poses both technical challenges, with up to 30% of biopsies providing insufficient sampling or false negative rates, as well as risk of complications from invasive sampling.^10^, ^11^, ^12^ In recent years, there has been a paradigm shift in the approach to the management of secondary liver malignancies, driven by the emergence of liquid biopsy‐based technologies, notably circulating tumor DNA (ctDNA) analysis. The utilization of ctDNA in the context of secondary liver malignancies holds immense promise for enhancing clinical decision‐making and patient outcomes as it provides insight into the particular biology of the metastatic disease.

In this narrative review we aim to summarize the existing literature on the applications of ctDNA in secondary liver malignancies and discuss future directions this technology to assist clinicians and researchers in clinical decision‐making and composing clinical studies for patients with secondary liver malignancies.

## 2 CIRCULATING TUMOR DNA

ctDNA is composed of short segments of tumor‐derived genetic material that is freely circulating in serum, urine, saliva, feces and bile.^13^, ^14^ The exact mechanism for the release of ctDNA into systemic circulation is not fully known, however, there are a few leading theories as to how it becomes disseminated. Apoptotic cell death, characterized by programmed cellular dismantling, results in the release of intact DNA fragments into circulation.^15^ Necrotic cell death, on the other hand, leads to the passive release of fragmented DNA due to cellular disruption and membrane permeabilization.^15^ In addition to these passive forms of tumor cells releasing ctDNA, there is emerging evidence to suggest tumors have active secretion mechanisms, such as exosome‐mediated release, which may contribute to the shedding of ctDNA into the bloodstream.^16^ Tumor exosomes can traverse the systemic circulation and deliver ctDNA, to distant sites, where they may influence the tumor microenvironment and facilitate metastatic spread of disease.

CtDNA analysis encompasses two distinct approaches: tumor‐informed and tumor‐agnostic analysis. Tumor‐informed analysis involves the identification and profiling of specific genetic alterations known to be present in the primary tumor or metastatic lesions.^17^ This approach relies on prior knowledge of the tumor's genomic landscape, such as somatic mutations, copy number alterations, and chromosomal rearrangements. By targeting tumor‐specific alterations, tumor‐informed analysis enables the detection of minimal residual disease, assessment of treatment response, and monitoring of disease recurrence.^17^, ^18^, ^19^ In contrast, tumor‐agnostic analysis entails a broader assessment of ctDNA without prior knowledge of the tumor's genetic makeup. This approach leverages next‐generation sequencing (NGS) technologies to evaluate entire ctDNA landscape, including somatic mutations, copy number variations, and epigenetic modifications. Tumor‐agnostic analysis holds promise for identifying novel biomarkers, uncovering rare genetic alterations, and elucidating tumor heterogeneity across different cancer types.

ctDNA has a short half‐life which allows it to serve as a near‐real time window into the biology of the patient's cancer.^20^, ^21^, ^22^, ^23^, ^24^ Advancements in NGS technologies, coupled with the development of robust bioinformatics tools, have paved the way for high‐throughput and sensitive detection of ctDNA alterations, thus posing it as a feasible option to sensitively detect.^13^, ^19^, ^20^, ^25^, ^26^

## 3 COLORECTAL CANCER LIVER METASTASIS

Colorectal cancer is the third most common malignancy among men and women and is the second deadliest malignancy overall.^27^, ^28^ CRLM is the most common cause of liver metastasis and can occur in 30%–50% of patients with colorectal cancer.^4^, ^29^, ^30^ While screening for colon cancer has well documented benefits, and early disease portends great 10‐year survival, CRLM has an estimated 10‐year survival of 5%.^4^, ^29^ Given the high risk of mortality from metastatic disease, there has been a large focus placed on detection of metastatic disease, tailored therapeutic intervention and surveillance of progression for which ctDNA has been utilized.

ctDNA has shown to be a very accurate test in the detection of non‐metastatic colorectal cancer, with sensitivity and specificity reaching 93% and 90%, respectively, and a variety of genetic mutations have been sited in CRLM (Table 1).^49^ Bessa et al. analyzed 623 blood samples from patients with colorectal cancer and stratified the sensitivity of ctDNA based off of TMN staging, showing sensitivities of 84% (Stage I), 94% (Stage II) and 96% (Stage III).^49^

**Table 1:** Summary of genetic pathways seen in CCLM and the corresponding clinical relevance.

Gene/genetic pathway	Study	ctDNA positivity	Relevance
APC	[31, 32, 33]	Some mutations	− Mutation in this gene affects tumor growth suppression. − Seen in 76% of CCLM patients. − Portends a worse disease‐free survival rate in patients receiving neoadjuvant therapy and liver resection.
TP53	[32, 34, 35, 36, 37]	Yes	− Mutation in this gene affects tumor proliferation. − Seen in 36%–74% of CCLM patients. − Isolated mutations in TP53 do not predict worse disease‐free survival.
KRAS	[34, 38, 39, 40, 41]	Yes	− Mutations in this gene affect cell signaling pathways which affect cell growth, maturation, and apoptosis. − Seen in 38% of CCLM patients. − Strong association with poor prognosis and short disease‐free recurrence − Also associated with a high tendency for recurrence and progression to peritoneal, bone, or brain dissemination. − Chemotherapy with Cetuximab results in high response rates for KRAS mutations.
RAS	[38, 40, 41, 42, 43]	Yes	− Mutations in this gene were seen in 25%–52% of CCLM patients. − Associated with more frequent positive resection margin. − Bevacizumab regimens produced an improved pathological response compared to cetuximab without notable disease‐free survival differences for RAS mutations.
BRAF	[38, 44, 45, 46, 47, 48]	Yes	− Mutation in this gene affects cell division signaling pathways. − Seen in 0%–9.1% of CCLM patients however likely affected by these patients presented with advanced unresectable disease, and incidence could be higher. − Associated with aggressive disease with overall poor outcomes, and within the first year, was a stronger predictor than R1 resections.

*Note*: Studies solely identifying colorectal cancer ctDNA without metastasis is not included here.

With the high level of sensitivity, and ability to provide real‐time insight into a patient's tumor biology, ctDNA has also been shown as useful in surveillance of CRLM.^50^, ^51^, ^52^, ^53^, ^54^ When examining patients who had undergone surgical resection, Chan et al. found that incorporation of longitudinal ctDNA analysis at 6‐months intervals improved liquid biopsy detection of disease with a sensitivity of 100% with others also supporting use of ctDNA for interval surveillance.^53^ This impact has already been integrated in some clinical practice such as at the Cleveland Clinic Foundation where the current protocol is to obtain ctDNA within 30 days of starting and completing any new chemotherapy treatment course, as well as within 30 days preoperatively and 30‐60 days postoperatively.^55^ Furthermore, the Cleveland group (Wehrle et al.) has demonstrated that post‐resection ctDNA positivity predicts recurrence in patients undergoing resection and transplant for CRLM.^55^ This highlights the utility of ctDNA in surveillance, and theoretically proposes utility in guiding adjuvant therapy. Both of these proposed uses have not otherwise been demonstrated definitively in CRLM but have been clearly shown in CRC in the 2023 DYNAMIC Trial and GALAXY study of the same year.^51^, ^56^, ^57^ Finally, though not a common treatment approach, ctDNA has been described by Wehrle et al before and after liver transplantation for CRLM, specifically showing that transplant can clear ctDNA in this context, and that postoperative ctDNA may help indicate recurrence, though the latter has not been described definitively.^58^

This level of sensitivity in post resection patients can be critical as recurrence holds a poor overall prognosis. Thus, improved guidance for and revision of adjuvant treatment would be optimal. When examining patients with CRLM status‐post resection, Tie et al. found that post resection ctDNA levels detected immediately after surgery had a recurrence risk of 83% which was significantly increased from patients with no ctDNA detected initially (31%).^52^, ^56^ Of these patients who had ctDNA detected postoperative who were unable to clear ctDNA after adjuvant therapy, all experienced recurrence of disease. Additionally, some research has shown that the particular genetic anomalies predicts a higher preponderance to recur in specific anatomic locations which may also provide an avenue to tailor patient surveillance measures.^38^ The IMPROVE‐IT2 trial is currently seeking to answer if a ctDNA guided surveillance strategy can be used compared to the standard CT surveillance and to determine the effect of this approach on the proportion of patients receiving curative intent resection.^59^

The utilization of ctDNA in CRLM for precision therapy is a growing field. Tie et al reported in the landmark DYNAMIC trial that ctDNA‐guided adjuvant therapeutic regimen for stage II colon cancer can de‐escalate adjuvant chemotherapy without increased risk of recurrence in the ctDNA directed group.^52^, ^56^ This has large implications in tailoring clinical therapies to optimize patient's care.^52^, ^56^ This personalized treatment may also improve overall utilization of healthcare system resources which is important to consider. Extensive research is currently underway regarding the use of ctDNA in guiding therapy for colorectal and CRLM, including the DYNAMIC‐III, VEGA, TRACC trials, each assessing ctDNA‐guided adjuvant chemotherapy.^59^, ^60^, ^61^, ^62^

## 4 BREAST CANCER

Breast cancer is the leading cancer for women in the United States.^63^, ^64^ While predominantly breast cancer is found to have local disease, 6% of disease is diagnosed with metastatic disease with a 5 year prognosis as poor as 28%.^63^, ^65^, ^66^ This level of poor prognosis highlights the need for sensitive and specific biomarkers for early detection and monitoring of metastatic disease. Studies have demonstrated the potential of ctDNA analysis in breast cancer liver metastases, showing sensitivities ranging from 75% to 90% and specificities exceeding 90%.^67^ Key genetic mutations detected in ctDNA include mutations in the PIK3CA, TP53, and ESR1 genes, among others.^67^ Riva Et al. looked at ctDNA for patients who received neoadjuvant therapy and noted that all but one patient showed reduction in ctDNA levels.^68^, ^69^, ^70^ For the one patient who did not see a decrease in ctDNA levels during neoadjuvant therapy, they notably had progression of disease despite chemotherapy. This poses ctDNA as a valuable tool to detect treatment response and may serve to cater neoadjuvant therapies in patients as there is no other current biomarker to help guide in the therapeutic tailoring for breast cancer. Moving forward, further research is needed to validate the clinical utility of ctDNA in guiding therapeutic decisions and predicting treatment responses in breast cancer liver metastases.

## 5 LUNG CANCER

Lung cancer is one of the most prevalent cancers in the entire world and nearly one fifth of patients with nonsmall cell lung cancer will develop liver metastasis.^9^, ^71^ The use of ctDNA for lung cancer is a relatively well established when compared to other disease locations. The National Cancer Comprehensive Network guidelines outline the utilization of ctDNA testing when patients are not medically fit for tissue sampling or there is insufficiency of a tissue sample.^72^ Sensitivity and specificity data for ctDNA detection in lung cancer liver metastases vary across studies, with reported sensitivities ranging from 70% to 85% and specificities exceeding 90%.^73^, ^74^, ^75^ Commonly detected mutations in ctDNA include alterations in the EGFR, KRAS, and ALK genes, among others.^76^ Iams et al. found that when tissue sampling and ctDNA were done concurrently ctDNA 5.5% of patients were found to have actionable variants identified uniquely via ctDNA despite successful tissue sampling.^77^ Additionally, in the ACCELERATE trial, 23% of patients were able to initiate early therapy with serum ctDNA results before tissue results, with 12% of patients having actionable alterations identified only through plasma testing.^78^ Research has also shown that allelic frequency of plasma alterations is higher in cases with liver metastasis and ctDNA positivity in lung cancer patients corresponding to higher tumor burden.^79^, ^80^ Despite the robust evidence for ctDNA use in lung cancer, further studies are required to identify its specific utilization for novel therapeutic targets specifically in lung metastasis to the liver.

## 6 PANCREATIC CANCER

Pancreatic cancer is the fourth leading cause of cancer death in the United States with the 5 year survival rate quoted to be 5%–15%.^81^ More than half of pancreatic cancers metastasize to the liver, with the liver being the most common site of pancreatic metastesis. Patients with pancreatic liver metastases face limited treatment options and even worse prognoses. Given the low survivability and aggressive nature of pancreatic cancer, the data on ctDNA remains limited at this time. Sensitivity and specificity data for ctDNA detection in pancreatic cancer liver metastases are limited but show promising results, with reported sensitivities ranging from 65% to 80%.^82^ Commonly detected mutations in ctDNA include alterations in the KRAS, TP53, and SMAD4 genes.^83^, ^84^, ^85^ Of those patients with KRAS mutations, they were found to have a notably higher risk of liver related metastasis (78%) and therefore may provide prognostic information for initial diagnosis or for disease progression monitoring,^85^ While ctDNA detection in pancreatic cancer shows clinical promise, patient's prognosis with pancreatic cancer is so poor with few therapeutic options that ctDNA's utility for clinical intervention remains limited at this time, but should be considered for prognostication and may be an avenue for targeted therapy once current management improves.^86^

## 7 CONCLUSION

**Figure 1:**
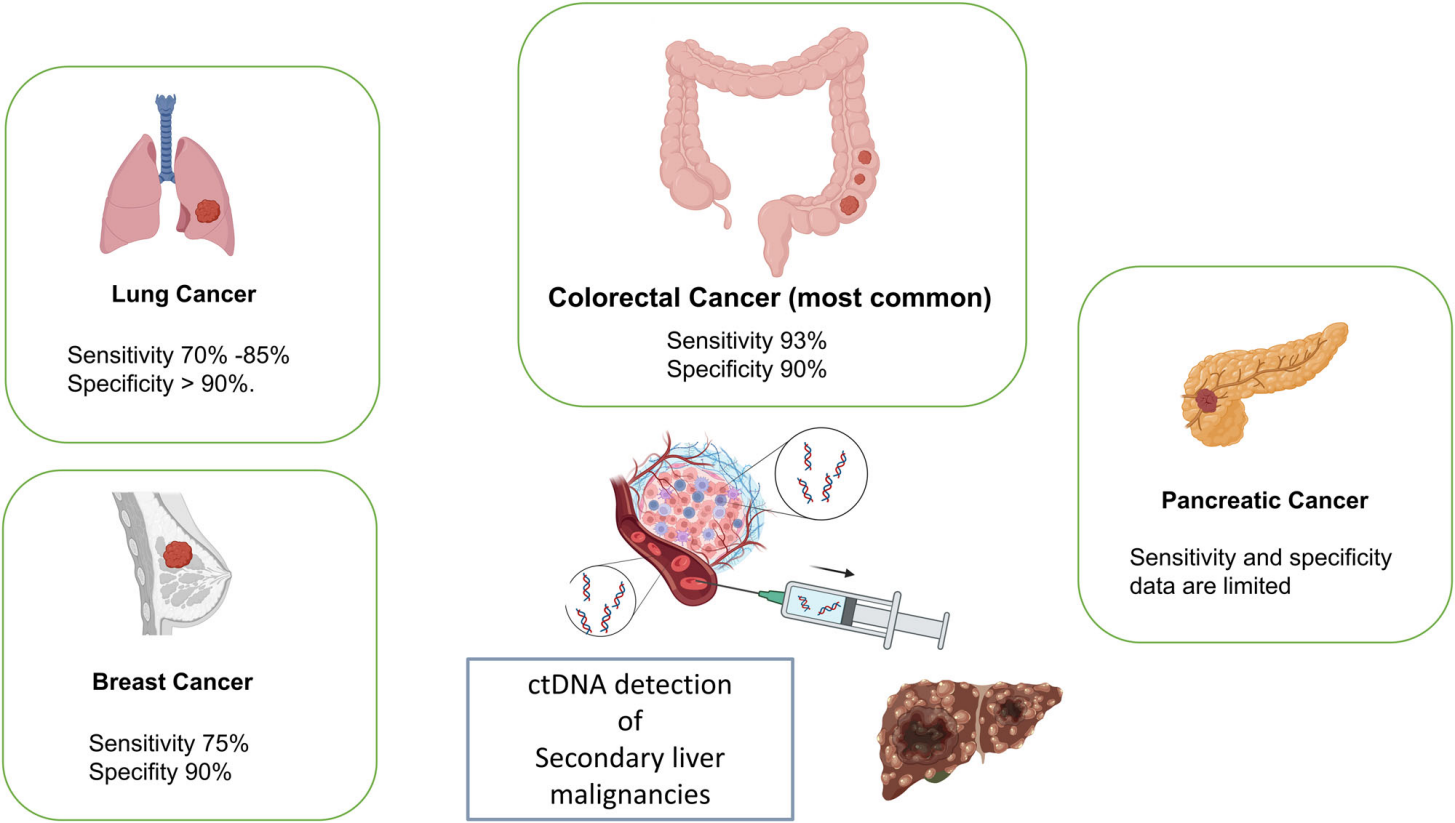
ctDNA in secondary liver malignancies.

ctDNA analysis holds promise for detecting liver metastases, guiding therapeutic targeting, and monitoring disease recurrence. By capturing the genetic heterogeneity of metastatic tumors, ctDNA analysis enables noninvasive assessment of tumor burden, identification of actionable genetic alterations, and monitoring of treatment response over time. When used concurrently with primary tissue sampling, ctDNA can provide a powerful tool for real‐time assessment of disease progression and emergence of treatment resistance. Future directions of ctDNA should aim at facilitating timely adjustments in therapeutic strategies to ensure personalized therapy based on tumor biology. Despite its relatively new use in many secondary liver malignancies, ctDNA remains an attractive option for serial sampling given its minimally invasive nature, and its promise in tracking disease response to therapy, or progression despite it. However, further research is needed to optimize ctDNA detection methods, validate its clinical utility, and identify novel therapeutic targets. Additionally, challenges such as standardization of assays, interpretation of results, and cost‐effectiveness need to be addressed to fully realize the potential of ctDNA in clinical practice (Figure 1).

## AUTHOR CONTRIBUTIONS

The study was conceptualized and conducted under the direction of Dr. Federico Aucejo and Dr. David Kwon. Literature review and analysis was performed by Noah X. Tocci, Chase J. Wehrle, and Hanna Hong. Manuscript drafting was performed by Noah X. Tocci, Chase J. Wehrle and Abby Gross. Critical manuscript review was performed by all authors.

## CONFLICT OF INTEREST STATEMENT

The authors declare no conflicts of interest.

## SYNOPSIS

Circulating tumor DNA (ctDNA) is emerging as a essential component in oncologic management. We review the utility of ctDNA in the management of secondary liver malignancies. This includes colorectal metastasis, in which there is the largest experience, as well as breast, lung, and pancreatic liver metastases.

## Data Availability

The data that support the findings of this study are available from the corresponding author upon reasonable request.
